# Performance of Older Persons in a Simulated Shopping Task Is Influenced by Priming with Age Stereotypes

**DOI:** 10.1371/journal.pone.0160739

**Published:** 2016-09-20

**Authors:** Otmar Bock, Selçuk Akpinar

**Affiliations:** 1 Institute of Physiology and Anatomy, German Sport University, Köln, Germany; 2 Department of Physical Education and Sport Education, Nevşehir Hacı Bektaş Veli University, Turkey; University of California, San Francisco, UNITED STATES

## Abstract

Previous research suggests that older persons show cognitive deficits in standardized laboratory tests, but not in more natural tests such as the Multiple Errands Task (MET). The absence of deficits in the latter tests has been attributed to the compensation of deficits by strategies based on life-long experience. To scrutinize this view, we primed older participants with positive or negative stereotypes about old age before administering MET. We found that compared to unprimed controls, priming with positive age stereotypes reduced the number of errors without changing response times, while priming with negative stereotypes changed neither errors not response times. We interpret our findings as evidence that positive age priming improved participants’ cognitive functions while leaving intact their experience-based compensation, and that negative age priming degraded participants’ cognitive functions which, however, was balanced by an even stronger experience-based compensation.

## Introduction

Among the most prominent signs of cognitive aging are deficits of executive functions and working memory [1]. Both are probably related to structural changes in the prefrontal cortex: this brain area shrinks in old age more dramatically than other brain regions do [2,3], exhibits a particularly prominent accumulation of senile plaques [4], and is functionally linked both with executive functions (review in [5]) and with working memory [6]. Stereotype Embodiment Theory [7, 8] offers an additional explanation of cognitive aging: older persons perform less well than young ones because they adopt and enact negative preconceptions about old age which prevail in society. On the positive side, this theory also stipulates that older persons will improve their performance after being exposed to *positive* preconceptions about old age; such preconceptions do exist, although they are less prevalent in many societies. Empirical support for Stereotype Embodiment Theory comes from studies on unconscious priming, where positive or negative age stereotypes were activated (‘primed’) without the participants’ awareness, such that participants could not willfully decide to counteract the activation. These studies have shown that persons exposed to negative age stereotypes subsequently perform worse on a range of motor and cognitive tasks than control participants, while persons exposed to positive age stereotypes perform better than control participants (see meta-analysis in [9]). The purpose of the present study is to evaluate whether the effects of age priming extend beyond standardized laboratory tasks, and include activities which resemble older persons’ everyday life.

There is a long-standing debate in literature about whether standardized laboratory tasks of cognitive and motor functions are indicative of a person’s cognitive and motor abilities in real life [10]. According to WHO terminology, standardized tasks determine the testees’ ‘capacity’, but not necessarily their everyday ‘performance’ [11]. It has been argued that standardized tasks differ from everyday activities since they are repetitive, externally instructed, draw attention, don’t serve a desirable ultimate goal, and don’t allow participants to compensate the deficit of one function by invoking alternative functions. These differences could explain why age-related cognitive [12] and motor [13, 14] deficits were distinctly different in standardized laboratory tasks and in realistic tasks. We reasoned that the effects of age priming might also be distinctly different in laboratory and in realistic tasks.

The realistic task selected for the present study is modeled after everyday shopping activities. Specifically, we developed a computer-based version of the Multiple Errands Task (MET; [15]), in which participants are instructed to walk through a pedestrian precinct and carry out “errands” such as to buy certain items, to gather information about other items, and to be in a given place at a given time. MET therefore mainly taps the participants’ memory and planning skills. Previous work has shown that MET scores discriminate patients with prefrontal lesions from age-matched controls [15], and are more sensitive to prefrontal dysfunction that traditional clinical tests [16]. MET therefore seems to be a valid predictor of prefrontal integrity. However, older participants were found to perform quite well on MET [17] and on similar realistic tasks [1,18], in spite of their deficits on standardized cognitive tests [5, 6]. This discrepancy has been interpreted as evidence that for realistic but not for laboratory-type tasks, healthy older persons can overcome their cognitive deficits by using workaround strategies acquired through life-long experience [1].

The proposed differential role of workaround strategies for realistic and for laboratory-type tasks could also be the basis for differential effects of age priming. Specifically, positive age primes may enhance cognitive functions and thus improve performance on both types of task; in contrast, negative age primes may reduce cognitive functions and thus degrade performance on standardized tasks, but may leave performance intact on realistic tasks where deficits are compensated by workaround strategies. Such a view implies that primes influence the cognitive processing of incoming information but not the use of strategies; since primes are thought to modify the transformation of *incoming stimuli* into attitudes and behavior [7, 19], it indeed remains conceivable that they don’t modify strategic compensation. Thus summing up, it is compatible with literature that for tasks like MET, positive age primes are more effective than negative ones, even though the opposite has been reported for their effects on laboratory tasks [9].

To examine out hypothesis, we have developed a portable MET variant which is not constrained to a given pedestrian precinct like the original version [15] nor to bulky and expensive virtual-reality setups like some modern versions [20], but rather can be implemented on any off-the-shelf laptop-PC, netbook or tablet. Portable MET variants have been used by others with success [18]. This approach allowed us to collect data at the participants’ home, where they may feel more comfortable and less intimidated than in a research laboratory.

## Methods

A total of 60 healthy persons voluntarily participated in this study. Participants were recruited and tested at Avanos, Nevsehir, Turkey. There were two criteria for the inclusion of the study, age and educational level. All the participants had to be between the age of 60 and 85, with at least a middle school educational level. They were randomly assigned to a negative (M_age_ = 69.2 and SD = 4.37), neutral (M_age_ = 68.05 and SD = 6.15), or positive (M_age_ = 68.15 and SD = 5.42) group for the purpose of priming, stratified by gender. Each group was comprised of 20 participants (10 female and 10 male). Five participants from the positive, five from the neutral and eight from the negative group were not regularly taking any medicine. In the positive group, five female and two male participants were regularly taking medicine for blood pressure, two male participants for diabetes, two male participants for prostate disease, and four male participants for heart disease. In the neutral group, four female and two male participants were regularly taking medicine for blood pressure, two male and three female participants for diabetes, two male participants for prostate disease, and one female and one male participant for heart disease. In the negative group, one male and two female participants were regularly taking medicine for blood pressure, one female and two male participants for diabetic disease, one male participant for prostate disease, and three female and two male participants for heart disease. All reported to have normal or corrected-to-normal vision, were native Turkish speakers and had secondary, high school, or university education levels. All participants had no prior knowledge as to the purpose of the study, and no experience with similar research. None of the participants had earlier experience with PC tablets but all reported that the tablet-based task was easy to perform. Written informed consent was obtained from each participant. The study was conducted in accordance with the Declaration of Helsinki as amended by the World Medical Association Declaration of Helsinki in 2013. It was pre-approved by the Ethics Committee of the German Sport University.

All participants were tested at home. They first completed a demographic questionnaire regarding their age, education level, number of people in their household, existing health problems, and the frequency of grocery shopping. Participants were then primed with age stereotypes using the scrambled sentence task [21]. In this task, each participant was given 20 cards of 5 words each, and had to pick four words from each card to formulate a meaningful sentence. Unbeknownst to the participants, one of the four selected words could be an age stereotype. For participants in the positive group, each card included one word reflecting a positive stereotype about old age (i.e., accomplished, active, alert, dignified, distinguished, etc.) whereas each card in the negative group had one word reflecting a negative stereotype about old age (i.e., impatience, unrespect, unsuccessful, unsociable, inexperienced, etc.). For participants in the neutral group, age stereotypes were absent. All participants completed the scrambled sentence task within less than 15 min.

The stereotype words were generated beforehand in the following way. Starting with five self-selected words denoting positive age stereotypes, the investigators compiled a list of 50 synonyms and then asked ten uninformed raters to rank-order all words according to how well they represent positive attitudes towards old age. Scores were then averaged across raters, and the highest-ranking words were used in the actual experiment. The same procedure was followed for negative stereotype words.

After the scrambled sentence task, the participants performed a virtual grocery shopping task modeled after MET [15]. This task was specifically developed for the current study, implemented on a 10-inch tablet PC with custom-made software, and imitated activities that might occur in a grocery store. There was no time restriction to do the task thus it was self-paced. First came an instruction screen, on which participants were told to purchase 4 different items, two of them with price limitations (e.g., tomatoes at less than 2.00 Lira). They were also told to remember the price of a fifth item, without purchasing it. Finally, they were told that an analog timer would start at the onset of shopping, and that they should touch the timer when 30 seconds elapsed. Our grocery shopping task therefore contained all challenges of the original MET (see Introduction).

**Fig 1:**
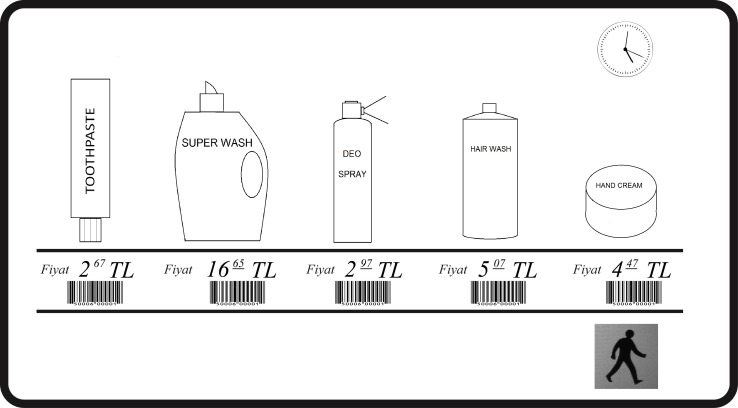
Illustration of our shopping task. An isle in the simulated grocery store is shown as it is presented to participants. It demonstrates the shopping items with prices, the clock, and the button for proceeding to the next screen. The products shown on this and on all other screens were familiar to all participants.

After reading the instructions, participants pressed the “start” button on the screen when they felt ready. This triggered the display of the analog timer in the upper-right corner, an aisle with photographs of five different grocery items above five realistic price labels, and an image of walking person that takes to the next isle (see Fig 1). Participants scanned the aisles for items to purchase and if they detected one, they “bought” it by tapping their finger on it. To advance to the next aisle, they tapped a button in the lower-right corner of the screen, showing the image of a walking person (see Fig 1; for brevity, this button is henceforth called “next” button). The next aisle with five items was then displayed, etc., for a total of ten isles and a total inventory of 50 items. Four of the aisles contained an item that should be purchased, but the order of those aisles didn’t match the order of items on the instruction screen. Two aisles contained an item that was priced too high, one aisle contained an item whose price should be remembered, and the remaining three aisles contained no relevant items. The last aisle was followed by a screen asking participants for the to-be-remembered price. Then came a new instruction screen with new grocery items and price limits and with a reminder to press the timer after 30 seconds, and eventually a third instruction screen with yet another set of grocery items and price limit and with yet another reminder to press the timer.

The tablet PC registered the time and location of button presses in the shopping task and, as an audio file, the verbal response for the to-be-remembered price. This allowed us to quantify three groups of variables, related to response timing, to response errors and to finger position variability. The former two groups are central to our hypothesis as they quantify MET performance; the latter group is secondary for the purposes of our study.

Time-related variables;

TN: time from pressing “next” to subsequently pressing “next” again
TB: time from pressing “next” to subsequently pressing “buy”
TC: time from pressing “next” to subsequently pressing the clock
TP: time from pressing “next” to subsequently starting to tell the remembered price
Error-related variables;
EN: error of not buying a required item
EB: error of buying a non-required item
EC: error of not pressing the clock within 30 s ±5 s
EA: error of not naming the correct to-be-remembered price
Finger position variability;
SX: standard deviation of finger presses about their button-specific mean in x (horizontal)
SY: standard deviation of finger presses about their button-specific mean in y (vertical)

The variables TN, TB, TC and TP were calculated as means across all instances, and the variables EN, EB, EC and EA as sums of all instances.

We additionally calculated three global variables to quantify the overall duration, errors and finger position variability:

GT: Duration of the complete experiment, minus the sum of all TC (such that participants who remembered to press the clock were not “punished” by a longer GT)
GE: Sum of all errors
GS: $\sqrt{\frac{{SX}^{2}+{SY}^{2}}{2}}$

After the priming task, participants completed the Tangible Support Scale (TSS), a subscale of the Interpersonal Support Evaluation List [22]. The TSS assesses the perceived availability of help with ten items on a four-level Likert scale, high scores indicating a high level of perceived support. Participants also completed the General Self-Efficacy Scale (GSE). Its original authors [23] and others have repeatedly modified the wording of GSE to assess self-efficacy for specific situations, and we modified it to assess self-efficacy for grocery shopping (e.g. “I am confident that I could deal with unexpected events in the grocery store”). As in earlier versions of this scale, responses were on a four-level Likert scale with high scores indicating a high level of confidence. TSS and GSE were introduced to control for individual differences of perceived self-efficacy before priming; they were used as co-variates in an ANCOVA (see below). Please note that TSS, and GSE were originally in English while scrambled sentence task and shopping task were originally developed in German; all verbal material had therefore to be translated into Turkish. Three native Turks confirmed that the words denoting positive and negative age stereotypes are adequate in Turkey, and that the products presented for shopping are commonly available in Turkey.

Dependent variables were submitted to analyses of variance (ANOVAs) with the between-participants factor Priming and the within-participants factor Repetition except for TSS and GSE, where Priming was the only factor. The global variables GT, GE and GS were submitted to analyses of Co-Variance (ANCOVAs) with the between-participants factor Priming and with the covariates Calendric Age, TSS and GSE.

## Results

**Fig 2:**
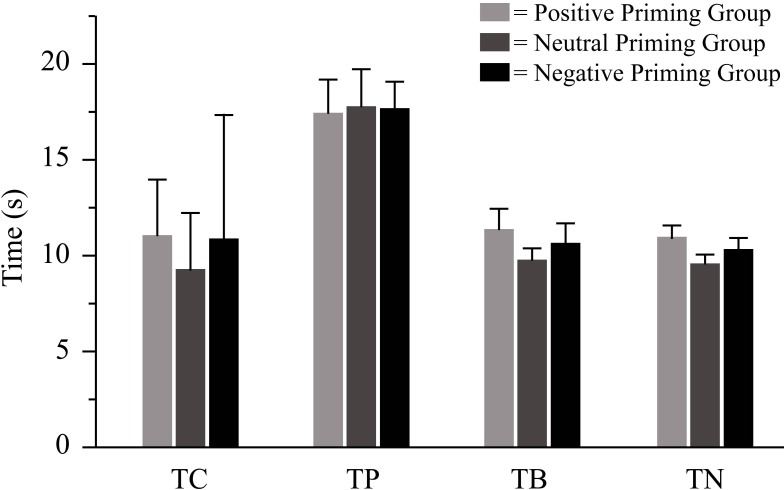
Effects of age priming on four variables related to shopping speed. For each variable, across-participant means and between-participant standard errors are plotted separately for each priming group. TN: time from pressing “next” to subsequently pressing “next” again, TB: time from pressing “next” to subsequently pressing “buy”, TC time from pressing “next” to subsequently pressing the clock, TP: time from pressing “next” to subsequently starting to tell the remembered price.

As Fig 2 illustrates, time-related variables differed from each other but were similar in all three priming groups. Accordingly, two-way ANOVA (see Table 1) yielded a non-significant main effect of Priming whereby time to perform the task (GT) was similar in the positive (M = 8.82 min, SD = 1.91 min), neutral (M = 7.97 min, SD = 1.68 min) and negative (M = 8.43 min, SD = 2.40 min) group. Likewise, Priming*Repetition was not significant, but significance emerged for Repetition as shown in Fig 2. The absence of priming effects on time-related variables is not a support for our hypothesis about the effects of age primes on MET performance (see Introduction).

**Table 1:** Influence of age priming on dependent variables related to time, errors and finger variability.^1^

	Priming	Repetition	Priming*Repetition
Time-related variables	F(2,18) = 0.034^n.s.^	F(3,54) = 8.15***	F(6,54) = 0.69 ^n.s.^
Error-related variables	F(2,57) = 18.32***	F(3,171) = 55.29***	F(6,171) = 5.86***
Finger variability- related variables	F(2,57) = 3.62*	F(1,57) = 119.71***	F(2,57) = 1.55 ^n.s.^

^1^Rows represent dependent variables, and columns different ANOVA-effects.

Numbers are F-values, with degrees of freedom in parentheses. ^n.s.^

* and

*** stand for p>0.05, p<0.05 and p<0.001, respectively.

The analysis of time-related variables is limited to participants who tapped the clock at least once and thus provided a data point for clock pressing time (TC); when TC was discarded TC and the remaining three variables were analyzed in all subjects, the significance pattern didn’t change.

**Fig 3:**
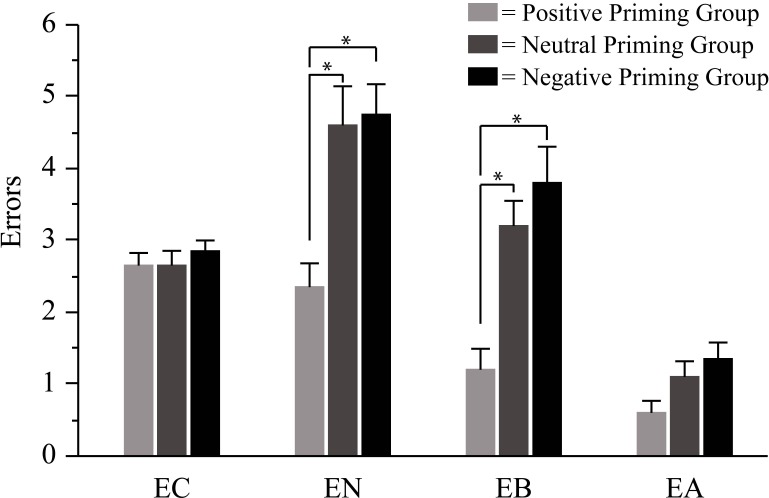
Effects of age priming on four variables related to shopping errors. For each variable, across-participant means and between-participant standard errors are plotted separately for each priming group. EN: error of not buying a required item, EB: error of buying a non-required item, EC: error of not pressing the clock within 30 s ±5 s, EA: error of not naming the correct to-be-remembered price. * = p < 0.01.

Fig 3 illustrates that error-related variables differed between priming groups, and two-way ANOVA accordingly revealed significance of Priming and Priming*Repetition (see Table 1). Post-hoc decomposition with Tukey’s HSD tests documented that only EN and EB differed between priming groups, and both differed only between the positive group on one side and the neutral and negative groups on the other side (EN: M = 2.35, SD = 1.38 for positive, M = 4.6, SD = 2.57 for neutral, M = 4.75, SD = 1.83 for negative group; EB: M = 1.2, SD = 1.15 for positive, M = 3.2, SD = 1.43 for neutral, M = 3.8, SD = 2.19 for negative group; p>0.05 for comparing neutral with negative group, p<0.01 for comparing positive group with neutral and with negative group).

Two-way ANOVA for finger position variability (see Table 1) yielded significance of Priming, with Tukey’s HSD tests confirming only a difference between positive and neutral group (p<0.05). Significance of Repetition emerged as well, since SY was larger than SX. No effects of Priming were obtained in one-way ANOVAs of TSS (F(2,57) = 1.23; p>0.05) and of GSE (F(2,57) = 1.51; p>0.05). These are secondary results, which neither confirm nor oppose our hypothesis (see Introduction).

Table 2 summarizes the ANCOVA outcome. When GT served as the dependent variable, only Calendric Age became significant; we explored the role of Calendric Age in a subsequent regression analysis, which found GT to increase on the average by 8.58 s per year (r = 0.38). When GE served as the dependent variable, significance emerged only for Priming whereby the overall error count (GE) was smaller in the positive group (M = 6.80, SD = 2.02) compared to the neutral (M = 12.75, SD = 3.92) and to the negative group (M = 11.55, SD = 3.61). With GS as the dependent variable, only GSE was significant; a subsequent regression analysis revealed that as self-efficacy increased, position variability decreased (r = - 0.39). Thus, the significant effect of Priming on position variability found in the above two-way ANOVA disappeared when self-efficacy was taken into account by an ANCOVA. Again, the result regarding GT is not a support of our hypothesis, the result regarding GE renews support for that hypothesis after controlling for co-variates, and the result regarding GS has no immediate bearing on the hypothesis.

**Table 2:** Influence of age priming on global scores for shopping time, errors and finger variability. Age, TSS- and GSE-scores were treated a co-variates in these analyses.^1^

	Priming	Age	TSS	GSE
GT	F(2,54) = 1.35^n.s.^	F(1,54) = 11.45**	F(1,54) = 0.02 ^n.s.^	F(1,54) = 1.94 ^n.s.^
GE	F(2,54) = 17.62***	F(1,54) = 0.04 ^n.s.^	F(1,54) = 0.61 ^n.s.^	F(1,54) = 0.64 ^n.s.^
GS	F(2,54) = 2.23 ^n.s.^	F(1,54) = 0.06 ^n.s.^	F(1,54) = 0.96 ^n.s.^	F(1,54) = 8.70**

^**1**^Rows, columns and cell entries have the same conventions as in Table 1.

GT: Duration of the complete experiment, GE: Sum of all errors, GS: Standard deviation of the finger position about the button center, TSS: Tangible support scale, GSE: General self-efficacy scale.

## Discussion

Our study evaluated the performance of older persons in a tablet-PC version of the Multiple Errands Task (MET). According to previous work, performance on that task is well preserved in older age (see Introduction) but we nevertheless observed an improvement when participants were primed with positive age stereotypes. In contrast, we found no decrements when participants were primed with negative age stereotypes. This outcome is only in part compatible with earlier research on age priming: a meta-analysis concluded that “effects from negative age priming were almost three times larger than those of positive priming when compared with a neutral baseline.” [9].

We have two possible interpretations for the unusual strength of positive compared to negative priming in our study. According to one, negative age priming was less effective in our study since performance was assessed with a realistic task rather than with a typical laboratory task; the cognitive deterioration induced in the present study could therefore be counteracted by a more pronounced use of compensatory strategies ([1], see Introduction). Our second interpretation draws on the fact that Turks have a more positive attitude towards old age than most other Europeans, as documented by attitude surveys [24] and seniors’ suicide rates [25]. When older Turks were asked about their expectations regarding old age, only 13.3% voiced negative attitudes, 84% positive attitudes, and 2.7% very positive attitudes [26]. It therefore is conceivable that Turks, being convinced that old age is venerable, are not easily persuaded that old age actually is lamentable. Both interpretations are tentative, and should be scrutinized by further research. The first explanation could be addressed by comparing the performance of the same participants, after priming with the same words, once in a typical laboratory task and once in a more realistic task. The second explanation could be evaluated by replicating the present study in a country where attitudes towards old age are less positive, and participants therefore are less well protected against negative age stereotypes.

In any case, our data are in line with earlier evidence showing that priming with positive age stereotypes is a robust technique [9]. In the past, this technique was found to improve older participants’ locomotion [27], handwriting [28], oculomotor learning [29], memory [30] and job performance [31] while reducing their stress level [32]; we now show that positive age priming also improves performance on a variant of the MET, a valid indicator of executive dysfunction [15,16,33].

It is interesting to note that positive age priming had no effect on performance speed, although speed is well known to decay with age and reliably did so even in our age-limited sample. Rather, priming had an effect on the error rate, as the positive group less often forgot to purchase a product and less often purchased a wrong product. Possibly, benefits of priming were quite specific and improved only the participants’ ability to keep a memorized shopping list up to date as they “walked” through the store and purchased item after item. This ability could correspond to the executive function ‘updating’, which includes the replacement of outdated information from working memory [34], but also to the executive function ‘inhibition’, which refers to the suppression of undesirable responses [5], such as purchasing an item that looks familiar or desirable but is not on the current shopping list.

The effects of priming on grocery shopping were not associated with noticeable effects on self-assessments of available help (TSS) and self-efficacy (GSE). This might seem surprising, in particular since GSE was adapted to capture the self-efficacy specifically for grocery shopping. However, we observed similar dissociated effects of age priming on performance but not on self-assessments in other experiments as well [31], and we therefore conjecture that benefits delivered below the level of consciousness–by the scrambled sentence task–are not accessible to self-rating which, necessarily, relies on awareness. However, we did observe an association between self-efficacy and finger position variability: as in earlier work [35], participants with higher levels of self-efficacy were less variable in their finger positions; the direction of causality in this association needs to be established by future studies.
